# An underlying diagnosis of osteonecrosis of bone is associated with worse outcomes than osteoarthritis after total hip arthroplasty

**DOI:** 10.1186/s12891-016-1385-0

**Published:** 2017-01-09

**Authors:** Jasvinder A. Singh, Jason Chen, Maria C. S. Inacio, Robert S. Namba, Elizabeth W. Paxton

**Affiliations:** 1 Medicine Service, Birmingham VA Medical Center, Birmingham, AL USA; 2 Department of Medicine at School of Medicine, and Division of Epidemiology at School of Public Health, University of Alabama, Faculty Office Tower 805B, 510 20th Street S, Birmingham, AL 35294 USA; 3 Department of Orthopedic Surgery, Mayo Clinic College of Medicine, Rochester, MN USA; 4 Department of Surgical Outcomes and Analysis, Kaiser Permanente, San Diego, CA USA; 5 Department of Orthopaedic Surgery, Kaiser Permanente, Irvine, CA USA

**Keywords:** Total hip replacement, Readmission, Osteoarthritis, Osteonecrosis, Arthroplasty, Joint replacement, Diagnosis, Risk factor

## Abstract

**Background:**

Well-designed studies of complications and readmission rates in patients undergoing total hip arthroplasty (THA) with osteonecrosis are lacking. Our objective was to examine if a diagnosis of osteonecrosis was associated with complications, mortality and readmission rates after THA.

**Methods:**

We analyzed prospectively collected data from an integrated healthcare system’s Total Joint Replacement Registry of adults with osteonecrosis vs. osteoarthritis (OA) undergoing unilateral primary THA during 2001–2012, in an observational cohort study. We examined mortality (90-day), revision (ever), deep (1 year) and superficial (30-day) surgical site infection (SSI), venous thromboembolism (VTE, 90-day), and unplanned readmission (90-day). Age, gender, race, body mass index, American Society of Anesthesiologists class, and diabetes were evaluated as confounders. We used logistic or Cox regression to calculate odds or hazard ratios (OR, HR) with 95% confidence intervals (CI).

**Results:**

Of the 47,523 primary THA cases, 45,252 (95.2%) had OA, and 2,271 (4.8%) had osteonecrosis. Compared to the OA, patients with osteonecrosis were younger (median age 55 vs. 67 years), and were less likely to be female (42.5% vs. 58.3%) or White (59.8% vs. 77.4%). Compared to the OA, the osteonecrosis cohort had higher crude incidence of 90-day mortality (0.7% vs. 0.3%), SSI (1.2% vs. 0.8%), unplanned readmission (9.6% vs. 5.2%) and revision (3.1% vs. 2.4%). After multivariable-adjustment, patients with osteonecrosis had a higher odds/hazard of mortality (OR: 2.48; 95% CI:1.31–4.72), SSI (OR: 1.67, 95%CI:1.11–2.51), unplanned 90-day readmissions (OR: 2.20; 95% CI:1.67–2.91) and a trend towards higher revision rate 1-year post-THA (HR: 1.32; 95% CI: 0.94–1.84), than OA patients.

**Conclusions:**

Compared to OA, a diagnosis of osteonecrosis was associated with worse outcomes post-THA. A detailed preoperative discussion including the risk of complications is needed for informed consent from patients with osteonecrosis.

**Electronic supplementary material:**

The online version of this article (doi:10.1186/s12891-016-1385-0) contains supplementary material, which is available to authorized users.

## Background

Total hip arthroplasty (THA) is a successful surgical treatment for end stage arthritis [1]. The most common underlying diagnosis in patients undergoing elective THA is osteoarthritis (OA), followed by osteonecrosis, congenital hip disorders, and inflammatory arthritis [2]. A systematic review of 67 studies with 2,593 patients with 3,277 hips concluded that osteonecrosis was not associated with higher revision rates after THA compared to the other underlying diagnoses [3], with minor exceptions. A small single-site retrospective study of 31 patients with glucocorticoid-induced osteonecrosis undergoing THA reported improvement in pain and function with THA but high complication and reoperation rates [4]; however, there were no controls.

Post-THA complications and associated hospital readmissions are undesirable THA outcomes of great interest clinically, due to associated morbidity. Post-arthroplasty complications, such as surgical site infections (SSI) and thromboembolic complications, are responsible for most surgically related readmissions [5–7]. Overall, 90-day readmission rates range 7–8% after primary THA or primary total knee arthroplasty [7–9]. Readmission rate can have a significant impact on health care resources, especially given the high volume of these procedures. Mortality is rare after THA (mostly elective) and is extremely undesirable.

We recently showed that an underlying diagnosis of RA was associated with higher readmission rate compared to OA [10]. To our knowledge, studies of complications and readmission rates in patients undergoing THA with osteonecrosis are lacking. It is possible that outcomes in patients with osteonecrosis undergoing THA have improved over time due to the improvement in bearing surfaces and ubiquitous use of cementless fixation [11].

The objective of our study was to examine whether compared to OA, an underlying diagnosis of osteonecrosis was associated with a higher adjusted risk of complications and readmissions after primary THA (study hypothesis). We also performed exploratory analyses to assess the association of the cause of osteonecrosis with complications and readmissions post-primary THA.

## Methods

We described study methods and results as recommended in the Strengthening of Reporting in Observational studies in Epidemiology (STROBE) statement [12].

### Study design, data source, and patient sample

We performed a cohort study of patients who had undergone an elective unilateral primary THA using the Kaiser Permanente (KP) Total Joint Replacement Registry (TJRR). We identified a cohort of patients who underwent elective unilateral primary THA between 04/01/2001 and 12/31/2012. The KP is a integrated healthcare system that covers over 9.5 million members in 7 US geographical areas. We have previously reported details on the KP TJRR data collection procedures, structure, and participation [13, 14]. The KP TJRR uses paper and electronic data capture tools to collect information by surgeons and other healthcare providers at the point of care, as well as data from other databases (including the electronic medical records) using electronic screening algorithms. The information from different data sources of the registry is linked using the integrated healthcare system’s unique patient identifiers. The data repository for the registry is a SQL database. A quarterly quality control check of these data ensures high accuracy. Specifically, the KP TJRR prospectively collects outcomes (i.e. readmissions, revision, SSI, venous thromboembolism (VTE)). Several outcomes including revision, SSI and VTE are adjudicated by review of charts by trained research associates [13, 14].

In a chart review of random sample of osteonecrosis and OA cases (*n* = 60; 30 cases each of osteonecrosis and OA) performed by an abstractor blinded to the database diagnosis, we found that the true positive rate of diagnoses (OA vs. osteonecrosis) was 88%. All registered cases in the KP TJRR are tracked until outcome and/or end of their lives. The voluntary participation of the KP TJRR in 2011 was 95% [14].

This study included all unilateral primary elective THA procedures, in patients aged 18 years old or older, with surgery indication for OA and/or osteonecrosis registered during the study period (*N* = 47,523). Patients who had a diagnosis of both OA and osteonecrosis (*N* = 563) were not included in the study. Patients who underwent revision THA, partial THA, or conversion procedures, as the index procedure, were not included in the study. This was done for two reasons: (1) the majority of THA are elective primary THA, our condition of interest; and (2) to keep the population homogeneous and allow easy interpretation of results. Cases from the geographical regions where the organization owns the hospitals (Southern California, Northern California, and Hawaii), covering 92% of the KP TJRR registered cases, were included. The final sample had cases from 50 hospitals and 362 surgeons.

### Outcome measures

The outcomes of interest in this study were mortality within 90 days, SSI (30 days for superficial and 1 year for deep infections), VTE within 90 days, unplanned readmissions within 90 days of the index primary elective THA, and revision (ever and for any reason). All outcome measures were obtained from the KP TJRR using 2001–12 data; timing of outcome assessment was chosen based on clinical relevance, based on feedback from an expert surgeon (R.N.). SSIs, which can be either deep or superficial were defined according to the Centers for Disease Control and Prevention/National Healthcare Safety Network criteria [15]. Superficial and deep SSI were also combined into one measure in this study since we anticipated a small number of events for these two components individually. The VTE measure, which included symptomatic deep vein thrombosis (DVT) and pulmonary embolism (PE) in this study due to the small number of events, were identified using the Agency for Healthcare Research and Quality patient safety indicators technical specifications [16] and further confirmed by chart review validation by trained clinical research associates. Readmissions are captured by the KP TJRR using quarterly extracts from the institutional electronic medical records of all hospital readmissions post discharge from the original joint arthroplasty procedure, available from 2009. Only unplanned readmissions, which were determined based on the 2014 Centers for Medicare and Medicaid Services Procedure-Specific Readmission Measures Updates and Specification Report for Elective Primary THA [17] were included in the analysis. Planned admissions such as those likely for subsequent hip arthroplasty procedures (e.g. International Classifications of Disease, 9th Revision, code (ICD-9) 715.34, Osteoarthrosis, localized, pelvic region and thigh and ICD-9: 733.42, aseptic necrosis of head and neck of femur) were not included in the reported readmissions. Revisions were defined as any re-operation of the index procedure where a component was replaced.

### Exposure of interest

An underlying diagnosis of osteonecrosis was the exposure of interest, compared with a diagnosis of OA. This information was obtained from the KP TJRR intra-operative forms completed by the surgeon at the time of surgery and does not rely on administrative data coding schemes. We also explored the associations by the cause/type of osteonecrosis, categorized as idiopathic vs. non-idiopathic, using the 2009–2012 data, the year of the beginning of prospective collection of intra-operative diagnosis and readmission data. Additional file 1 shows the sub-categories of non-idiopathic category glucocorticoid-induced vs. not glucocorticoid-induced (including hip fracture, alcohol abuse, sickle cell disease, lupus, pancreatitis, HIV, vasculitis and other autoimmune conditions).

### Covariates and potential confounders

Age (<65 and ≥65 years old, based on Medicare-eligible age of 65 years and higher), gender; race (White and Others), body mass index (BMI, continuous in 5 unit increment); American Society of Anesthesiologists (ASA) class (<3 vs. ≥3), and medical comorbidities using the Elixhauser co-morbidity measure [18] were evaluated as possible confounders/covariates.

### Statistical analyses

We calculated summary statistics to describe the study sample by whether the primary diagnosis was osteonecrosis vs. OA. We compared categorical variables between the two groups using 2-sided chi-square or Fisher’s exact tests where appropriate. A logistic regression was used to model the relationship between osteonecrosis (OA being the reference category) and mortality, VTE, SSIs, and readmissions while accounting for the nesting of observations within the surgeon variable. We created Cox proportional hazard models to evaluate the association of osteonecrosis compared to OA and the time to revision surgery. Covariates tested as confounders, were included in the final adjusted model if they changed estimates by >10% and *p* < 0.10 or deemed clinically important for the relationship studied. Odds ratios (OR), hazard ratios (HR), 95% confidence internals (CI), and *p*-values are reported. A sensitivity analysis was conducted to evaluate the robustness of estimations accounting for missing data. We also performed sensitivity analyses that adjusted for the competing risk of death. No formal sample size calculations were done for this observational cohort study. Data were analyzed using SAS (Version 9.2, SAS Institute, Cary, NC, USA) and *p*-value <0.05 was considered statistical significant.

## Results

### Demographic and clinical characteristics of study sample

There were 47,523 patients included in the study from 2001 to 12. Of these, 2,271 (4.8%) had an underlying diagnosis of osteonecrosis. Demographic and clinical features of patients with osteonecrosis are shown in Table 1. Compared to the cohort of patients with OA, the osteonecrosis group was younger (median (interquartile range (IQR)): 55 (46–64) vs. 67 (59–75)) years old, had more males (1,306 (57.5%) vs. 18,875 (41.7%)), and had less White (1,358 (59.8%) vs. 35,009 (77.4%)) patients. Patients with osteonecrosis had lower BMI (<30 kg/m^2^: 1,505 (66.3%) vs. 26,358 (58.3%)), slightly higher ASA scores (ASA ≥ 3: 897 (39.5%) vs. 15,827 (35.0%)), and were less likely to have diabetes (451 (19.9%) vs. 10,042 (22.2%)). Additional file 2 shows that several comorbidities were more prevalent in patients with osteonecrosis compared with those with osteoarthritis.

**Table 1:** Study cohort characteristics by primary diagnosis at total hip arthroplasty surgery

		Total		Osteoarthritis		Osteonecrosis	
		n	%	n	%	n	%
Total N		47,523	100.0	45,252	95.2	2,271	4.8
Age, years	Median (IQR)	66.0 (58.0–74.0)		67.0 (59.0–75.0)		55.0 (46.0–64.0)	
Age category, years	<65	20,899	44.0	19,195	42.4	1,704	75.0
	≥65	26,624	56.0	26,057	57.6	567	25.0
Gender	Male	20,181	42.5	18,875	41.7	1,306	57.5
	Female	27,341	57.5	26,376	58.3	965	42.5
Race	White	36,367	76.5	35,009	77.4	1,358	59.8
	Black	3,637	7.7	3,324	7.4	313	13.8
	Hispanic	3,283	6.9	3,030	6.7	253	11.1
	Asian	1,606	3.4	1,436	3.2	170	7.5
	Other/Multi-racial	753	1.6	707	1.6	46	2.0
	Unknown	1,877	4.0	1,746	3.9	131	5.8
BMI category^a^, kg/m^2^	<30	27,863	58.6	26,358	58.3	1,505	66.3
	≥30 and <35	11,625	24.5	11,170	24.7	455	20.0
	≥35	7,394	15.6	7,138	15.8	256	11.3
ASA category^a^	1 and 2	29,312	61.7	28,008	61.9	1,304	57.4
	≥3	16,724	35.2	15,827	35.0	897	39.5
Diabetes		10,493	22.1	10,042	22.2	451	19.9

*IQR* interquartile range, *BMI* body mass index, *ASA* American Society of Anesthesiologists

^a^Missing data: BMI (1.4%), ASA (3.1%)

Between 2009 and 2012 (period of prospective collection of intra-operative diagnosis and readmission data), of a total of 670 patients, 507 (76%) had idiopathic osteonecrosis and ​163 (24%) had non-idiopathic osteonecrosis. Among the non-idiopathic category, 40 (6%) patients had glucocorticoid-induced osteonecrosis and 123 (18%) patients had causes other than glucocorticoid-induced osteonecrosis. Additional file 3 shows that patient characteristics were similar between idiopathic vs. non-idiopathic etiology; similarly Additional file 4 shows that characteristics were similar between glucocorticoid-induced vs. not glucocorticoid-induced osteonecrosis.

### Crude outcome incidence

**Fig. 1:**
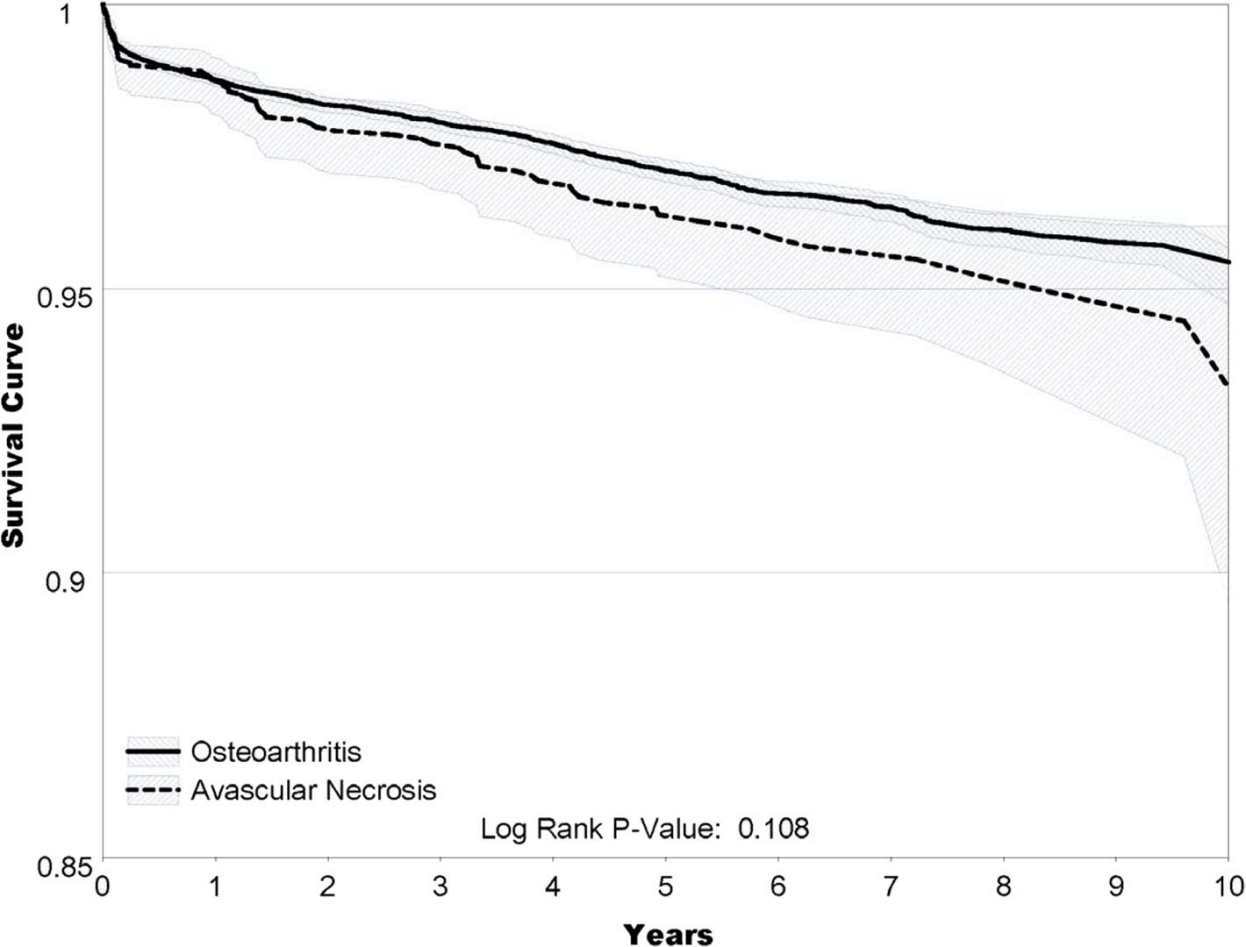
Revision Free Survival Estimates for Cohorts with Osteonecrosis and Osteoarthritis. The figure shows the hip implant survival curve comparing osteoarthritis to osteonecrosis after primary total hip arthroplasty. The unadjusted revision-free survival showed a trend favoring osteoarthritis compared to osteonecrosis (*p* = 0.108)

Compared to patients with OA, patients with osteonecrosis had higher incidence of the following complications post-THA: 90-day mortality (0.7% vs. 0.3%), SSI (combined: 1.2% vs. 0.8%; 90-day deep infection: 0.6% vs. 0.5%; 30-day superficial infection: 0.6% vs. 0.3%), 90-day VTE (1.4% vs. 1.2%, DVT: 0.8% vs. 0.7%, PE: 0.6% vs. 0.5%), 90-day unplanned readmission (9.6% vs. 5.2%) and revision at any time during the follow-up (3.1% vs. 2.4%; Table 2). The median follow up time for revision outcome was 3.2 years (Interquartile range, 1.4–5.8). Unadjusted revision-free survival showed a trend favoring osteoarthritis compared to osteonecrosis (Fig. 1; *p* = 0.108).

**Table 2:** Crude incidence of outcomes by primary diagnosis at total hip arthroplasty surgery

	Osteoarthritis		Osteonecrosis	
	n	%	n	%
Death, 90 days	138	0.3	15	0.7
Surgical site infection, any	367	0.8	27	1.2
Deep, 1-year^a^	230	0.5	13	0.6
Superficial, 30-days^b^	137	0.3	14	0.6
Venous thromboembolism, 90-days	551	1.2	31	1.4
Deep vein thrombosis^c^	323	0.7	18	0.8
Pulmonary embolism^d^	228	0.5	13	0.6
Readmission, 90 days unplanned^e^	846	5.2	64	9.6
Revision, ever^f^	1,075	2.4	70	3.1

^a^Deep surgical site infection excluded patient who died or left the system within 1 year without deep SSI (1549, 3.3%), *n* = 45974 (96.7%)

^b^Superficial surgical site infection excluded patient who died or left the system within 30 days without superficial SSI (138, 0.3%), *n* = 47385 (99.7%)

^c^Deep vein thrombosis (DVT) excluded patient who died or left the system within 90 days without DVT (376, 0.8%), *n* = 47147 (99.2%)

^d^Pulmonary embolism (PE) excluded patient who died or left the system within 90 days without PE (374, 0.8%), *n* = 47149 (99.2%)

^e^Readmissions includes limited sample (operative years 2009–2012 and 3 geographical regions), *n* = 17179. Excluded patient who died or left the system within 90 days without readmission (85, 0.5%), final *n* = 17094 (99.5%)

^f^The median follow-up for revision outcome was 3.2 years (inter-quartile range, 1.4–5.8 years)

Additional files 5 and 6 shows that the unadjusted rates of outcomes were similar by underlying cause of osteonecrosis except the following: higher 90-day readmission (14.7% vs. 7.7%) and revision rates (3.1% vs. 1.2%) in non-idiopathic group vs. idiopathic group (see Additional file 5); and higher 90-day readmission rate (17.5% vs. 13.8%) in glucocorticoid-induced vs. not glucocorticoid-induced osteonecrosis (see Additional file 6),

### Adjusted odds of mortality, SSI, VTE and readmission and adjusted risk of revision

After adjusting for age, race, BMI, and ASA class, the risk of 90-day mortality was significantly higher for patients with osteonecrosis than OA for the overall sample (OR: 2.48; 95% CI, 1.31–4.72) (Table 3). After adjusting for BMI, the odds of SSI (deep and superficial) in patients with osteonecrosis were 1.67 (95% CI: 1.11–2.51) times higher than patients with OA (Table 3). In the age-adjusted model, the risk of VTE (DVT and PE) was not statistically significantly different between two groups (OR: 1.37; 95% CI, 0.92–2.03) (Table 3). After adjusting for age, 90-day unplanned readmission was statistically significantly higher in patients with osteonecrosis than those with OA (OR: 2.20; 95% CI, 1.67–2.91) (Table 3).

**Table 3:** Unadjusted and adjusted association of osteonecrosis with death, surgical site infection (SSI), venous thromboembolism (VTE), and revision compared to osteoarthritis in patients undergoing total hip arthroplasty

Osteonecrosis vs. Osteoarthritis	Unadjusted OR (95%CI)	*p*-value	Adjusted OR (95%CI)	*p*-value
Death, 90 days^a^	2.12 (1.23–3.66)	0.007	2.48 (1.31–4.72)	0.006
SSI (deep and superficial)^b^	1.45 (0.97–2.18)	0.069	1.67 (1.11–2.51)	0.013
VTE (DVT and PE), <90 days^c^	1.16 (0.78–1.70)	0.466	1.37 (0.92–2.03)	0.118
Readmission, 90 days unplanned^d^	1.89 (1.43–2.48)	<0.001	2.20 (1.67–2.91)	<0.001
	Unadjusted HR (95%CI)	*p*-value	Adjusted HR (95%CI)	*p*-value
Revision (within 1 Year)^e^	1.00 (0.70–1.44)	0.992	0.92 (0.63–1.33)	0.652
Revision (after 1 Year)	1.44 (1.04–2.01)	0.030	1.32 (0.94–1.84)	0.109

*SSI* surgical site infection, as defined by the CDC, *VTE* venous thromboembolism, *DVT* deep vein thrombosis, *PE* pulmonary embolism- as defined by AHRQ, *OR* odds ratio, *HR* hazard ratio, *CI* confidence interval

All multivariable models were adjusted for covariates if they changed estimates by >10% and *p* < 0.10 in univariate analyses or deemed clinically important for the relationship studied

^a^Adjusted model excluded cases with missing BMI (641, 1.4%) and ASA (1,487, 3.1%), final *n* = 45,431 (95.6%). Multivariable-adjusted model was adjusted for age, race, BMI, and ASA

^b^The combined SSI includes 30-day superficial SSI and 1-year deep SSI. Adjusted model excluded cases with missing BMI (641, 1.4%), ad patients who died or left the system within 1 year (1,539, 3.2%), final *n* = 45506 (95.8%). Multivariable-adjusted model was adjusted for BMI

^c^The combined VTE outcome includes deep vein thrombosis and pulmonary embolism. Adjusted model excluded cases where patients either died or left the system within 90 days (*n* = 370, 0.8%), final *n* = 47,153 (99.2%). Multivariable-adjusted model was adjusted for age

^d^Readmissions includes limited sample (operative years 2009–2012 and 3 geographical regions), *n* = 17,179. Final adjusted model excluded cases where patients either died or left the system within 90 days (*n* = 85, 0.5%), final *n* = 17,094 (99.5%). Multivariable-adjusted model was adjusted for age

^e^Adjusted model had no exclusions due to missing values, *n* = 47,523 (100%). Multivariable-adjusted model was adjusted for age

A time interaction was observed for revision after adjusting for age. While the risk estimates for revision surgery were not statistically significant overall, the direction of the risk estimates changed for patients within one year of the surgery vs. longer time, for OA vs. osteonecrosis. Specifically, compared to patients with OA, patients with osteonecrosis did not have any significantly different hazard of revision (HR = 0.92, 95% CI: 0.63–1.33) within one year of the surgery, but after one year, patients with osteonecrosis seemed to have a 1.32-times higher (95% CI: 0.94–1.84) hazard of revision surgery (Table 3).

Exploring differences by the underlying cause of osteonecrosis, the age-adjusted 90-day readmission was significantly higher in non-idiopathic vs. idiopathic group, with adjusted odds ratio of 2.13 (95% CI: 1.23–3.68). Additional file 7 shows that the revision rate seemed higher, but did not meet statistical significance. Additional file 8 shows that there was no significant difference in 90-day readmission was noted in glucocorticoid-induced vs. not glucocorticoid-induced osteonecrosis.

### Reasons for readmission

The five most common principal discharge ICD-9 codes for the unplanned 90-day readmissions in patients with osteonecrosis were as follows: 996.42 (Dislocation of prosthetic joint), 038.9 (Unspecified septicemia), 486 (Pneumonia), 282.62 (Hemoglobin SS disease, sickle cell disease with crisis), and 493.22 (Chronic obstructive lung disease/asthma with exacerbation). In comparison, the five most common principal discharge ICD 9 codes for 90-day readmissions in patients with OA were as follows: 996.42 (dislocation of prosthetic joint), 996.66 (infection & inflammation reaction due to internal joint prosthesis), 998.59 (other postoperative infection), 38.9 (unspecified septicemia), and 996.44 (periprosthetic fracture around prosthetic joint). Additional file 9 shows the top 15 causes for readmission.

### Sensitivity analyses: missing data and competing risk of death

The impact of the 2,092 (4.4%) missing data (1,487 (3.1%) in ASA class, 1 (0.0%) in gender, and 641 (1.3%) in BMI) was evaluated. The distribution of other patient characteristics between patients with missing data and those with complete data were not different. The crude incidence rates for all outcomes were not statistically significantly different except for 90-day mortality (1.2% in patients with missing data vs. 0.3% others). To evaluate the impact of these missing data points in the estimations, we modeled diagnoses and 90-day mortality without BMI and ASA adjustments and found 90-day mortality for patients with osteonecrosis was increased (OR: 3.39; 95% CI, 1.93–5.96, *p* < 0.001), indicating that main analyses provided conservative estimates. Sensitivity analyses that adjusted for competing risk of death showed that there were no changes in any of the above analyses (data available on request).

## Discussion

In this study of 47,523 patients in KP TJRR registry undergoing primary THA, of whom 2,271 had osteonecrosis, we found that an underlying diagnosis of osteonecrosis was associated with higher risk of SSI, 90-day readmission and mortality following THA compared to OA. To our knowledge, our study is one of the most comprehensive studies to quantify the rate of several complications and unplanned 90-day readmission post-THA in a large, representative total joint registry study with validated outcomes. Our study overcomes a key limitation of previous studies, i.e., lack of a comparator group [3]. Our study also fills an important knowledge gap, i.e., are clinically important outcomes including revision surgery higher in patients with osteonecrosis? Several findings are novel and deserve further discussion.

The 90-day readmission rates following THA were 50% higher in patients with osteonecrosis compared to OA. To our knowledge, this is a novel finding of high interest to surgeons and policyholders. In absence of any previously published studies, this is the first study to demonstrate this difference. Readmission after an elective surgery such as THA can significantly influence health care utilization. Given that osteonecrosis is the second most common reason for primary THA, this excess utilization is clinically meaningful. With 332,000 THAs performed in 2010 in the US, the 90-day post-THA unplanned readmission rate of 6.8% translates into >12,000 admissions annually. Our finding of higher unplanned readmission rates in patients with osteonecrosis post-THA with an odds ratio of 2.20 adds to our recent study finding that RA was associated with 74% higher 90-day readmission compared to OA in patients undergoing THA or total knee arthroplasty [19].

We hypothesized the following potential reasons for a higher rate of readmission in patients with osteonecrosis compared to OA: (1) a higher rate of corticosteroid use or alcohol consumption, frequent causes of osteonecrosis (as noted by us, Additional file 1); (2) higher comorbidity and complexity in patients with osteonecrosis (higher ASA class in a previous study) [20]; (3) association of osteonecrosis with conditions such as trauma, autoimmune diseases and coagulation abnormalities, which are potential risk factors for poor THA outcomes; and (4) potential differences in bone structure and quality in osteonecrosis vs. OA, which might be related to implant-related complications. In fact, our data show that the prevalence of certain comorbidities (e.g. liver disease, chronic liver disease, deficiency anemia, alcohol abuse) was higher in patients with osteonecrosis.

The 90-day mortality was 2.5-times higher in patients with osteonecrosis who underwent primary THA, compared to OA. Due to the limitation of most previous studies of osteonecrosis to patients under the age of 50 years (and even smaller sample sizes), the ability to study a rare outcome, such as mortality, was previously limited. A large study sample size allowed us to assess this outcome appropriately. As noted above, a higher rate of comorbidity and potentially corticosteroid-associated complications in patients with osteonecrosis, may have contributed to higher mortality rates. Regardless of the cause, patients with osteonecrosis undergoing primary THA should be made aware of our finding of a significant difference in mortality post-THA compared to OA patients undergoing THA, providing them with relative and absolute risk. Based on our study findings, more studies into the etiology of this rare, but critical outcome are needed in patients with osteonecrosis. A better understanding of modifiable risk factors that are associated with excess mortality can allow them to be targeted for interventions aimed at reducing mortality in patients with osteonecrosis undergoing primary THA.

Another important and new clinical finding from our study was the higher risk of SSI in patients with osteonecrosis undergoing primary THA. Corticosteroid use, alcohol use or autoimmune diseases, risk factors for osteonecrosis, may also increase the risk of SSIs, due to known associations of these factors with the risk of infections [21–23]. One study of patients with glucocorticoid-induced osteonecrosis in patients undergoing THA without any controls showed a high complication rate [4]. Our study adds to this literature by showing that outcomes such as SSIs occurred more frequently in the overall cohort of patients with osteonecrosis undergoing primary THA, not just those with glucocorticoid-induced osteonecrosis.

In exploratory analyses by the underlying cause of osteonecrosis, adjusted 90-day readmission was significantly higher (2.13-times) and revision rate borderline higher, in non-idiopathic vs. idiopathic group. This is not surprising considering that comorbidities (alcohol abuse, lupus, hip fracture etc.) in the non-idiopathic are potential risk factors for poor outcomes and readmissions. Numbers for complications/outcomes by glucocorticoid-induced vs. other causes of osteonecrosis were too small to allow any meaningful comparison.

Our study has several limitations. Despite our efforts to control for several covariates and confounders in our observational study, residual confounding bias is possible. We were unable to investigate the outcomes of osteonecrosis by each underlying reason (corticosteroid, alcohol abuse, sickle cell disease, lupus etc.), due to the limitation of the number of patients with these diagnoses in our cohort, making the analyses very underpowered to perform any analyses. Larger studies in the future (perhaps data from national arthroplasty registries or data from several national registries combined) should investigate whether osteonecrosis associated with sickle cell, alcohol abuse etc. has worse outcome than other causes of osteonecrosis. These findings may not be generalizable to the U.S. general population; however, the membership of KP has been shown to be demographically and socio-economically representative of the geographical regions it covers [24, 25]. The study strengths include a large sample size, comparison of OA to osteonecrosis and the adjustment for important covariates. Some patients may have died before having one of the outcome measures because we treated each outcome as an independent event. Therefore, it is possible that we underestimated the incidence of the other complications. However, we performed models with death as the competing risk that showed no change in analyses, indicating that the associations of osteonecrosis we noted were not affected by the competing mortality risk.

## Conclusions

In summary, we found patients with osteonecrosis had higher risk of mortality, SSI and unplanned readmission post-THA, compared to patients with OA. These differences were both clinically meaningful and statistically significant. Our study findings have clinical implications. Surgeons can discuss increased rates of these complications/risks (absolute and relative) with patients with osteonecrosis before obtaining informed consent from patients undergoing THA. Our study also has implications for future research. We identified several aspects of poorer outcomes in patients with osteonecrosis compared to those with OA that need further investigation. Research into the underlying reasons for higher complication rate in patients with osteonecrosis undergoing primary THA should help understand this key clinical problem.

## Additional files

Additional file 1: Diagnostic codes for subtypes of osteonecrosis. This file shows the idiopathic and non-idiopathic causes of osteonecrosis, alongside the diagnostic codes and further sub-categorization of non-idiopathic osteonecrosis into glucocorticoid-induced vs. other causes. (DOCX 15 kb)
Additional file 2: Prevalence of Comorbidities by Primary Diagnosis at Total Hip Arthroplasty Surgery. This file shows the prevalence of various comorbidities in patients with osteoarthritis vs. osteonecrosis as the underlying reason and the primary diagnosis for the Total Hip Arthroplasty Surgery. (DOCX 20 kb)
Additional file 3: Characteristics of patients with osteonecrosis by the underlying etiology, Idiopathic vs. Non-Idiopathic. This file shows the comparison of key characteristics between Idiopathic and Non-Idiopathic etiology of osteonecrosis. (DOCX 18 kb)
Additional file 4: Patient characteristics for patients with Non-Idiopathic osteonecrosis only (Glucocorticoid-induced vs. Not). This file shows the comparison of key characteristics between Glucocorticoid-induced vs. Not glucocorticoid-induced among those with a non-Idiopathic etiology of osteonecrosis. (DOCX 16 kb)
Additional file 5: Unadjusted outcomes in patients with osteonecrosis by the underlying etiology, Idiopathic vs. Non-Idiopathic. This file shows the unadjusted comparisons of the outcomes in patients with Idiopathic vs. Non-Idiopathic etiology of osteonecrosis. (DOCX 15 kb)
Additional file 6: Unadjusted outcomes in patients with Non-Idiopathic osteonecrosis only (Glucocorticoid-induced vs. Not Glucocorticoid-induced). This file shows the unadjusted comparisons of the outcomes in patients with Glucocorticoid-induced vs. Not Glucocorticoid-induced etiology of osteonecrosis among those with a non-Idiopathic etiology of osteonecrosis. (DOCX 14 kb)
Additional file 7: Adjusted association of Non-Idiopathic vs. idiopathic cause of osteonecrosis with outcomes, revision and 90-day unplanned readmissions. This file shows the age-adjusted association of non-Idiopathic vs. idiopathic cause of osteonecrosis for revision and 90-day unplanned readmissions. (DOCX 14 kb)
Additional file 8: Adjusted* association of glucocorticoid-induced osteonecrosis vs. not glucocorticoid-induced osteonecrosis with outcomes. This file shows the age-adjusted association of glucocorticoid-induced osteonecrosis vs. not glucocorticoid-induced osteonecrosis for 90-day unplanned readmissions. (DOCX 14 kb)
Additional file 9: Top 15 International Classification of Diseases, Ninth Revision (ICD-9) codes and descriptions of unplanned readmissions within 90 days of Total Hip Arthroplasty Surgery by primary diagnosis (osteoarthritis vs. osteonecrosis). This file shows the top 15 reasons for unplanned readmissions within 90 days of Total Hip Arthroplasty Surgery by primary diagnosis, osteoarthritis vs. osteonecrosis. (DOCX 17 kb)

## Abbreviations

ASA: American Society of Anesthesiologists
BMI: Body mass index
CI: Confidence internals
HR: Hazard ratio
OA: Osteoarthritis
OR: Odds ratio
THA: Total hip arthroplasty
