# Supplementary material for: An underlying diagnosis of osteonecrosis of bone is associated with worse outcomes than osteoarthritis after total hip arthroplasty
Source: BMC Musculoskelet Disord. 2017 Jan 9;18:8. doi: 10.1186/s12891-016-1385-0 (PMC5223478; doi:10.1186/s12891-016-1385-0)
Supplement: Additional file 1: — Diagnostic codes for subtypes of osteonecrosis. This file shows the idiopathic and non-idiopathic causes of osteonecrosis, alongside the diagnostic codes and further sub-categorization of non-idiopathic osteonecrosis into glucocorticoid-induced vs. other causes. (DOCX 15 kb) [file 12891_2016_1385_MOESM1_ESM.docx]

**Additional file 1.** Diagnostic codes for subtypes of osteonecrosis

| Category | Description | Diagnostic code in Kaiser Permanente databases |
| --- | --- | --- |
| NON-IDIOPATHIC |  |  |
| Glucocorticoid-induced | Long term steroid use | V58.65 |
| Hip fracture associated | History of Trauma [fracture or dislocation of hip] | 820.x, 821.x, 835.x codes |
| Alcohol use related | Alcohol Abuse | 265.2, 291.1-291.3, 291.5-291.9, 303.0, 303.9, 305.0, 357.5, 425.5, 535.3, 571.0-571.3, 980.x, V11.3 |
| Lupus related | Lupus | 710.0 |
| Sickle cell disease related | Sickle cell anemia | 282.5 |
| Vasculitis related | Vasculitis | 447.6 |
| HIV related | HIV | 042 code |
| Pancreatitis related | Pancreatitis | 577.0, 577.1 |
| Other Autoimmune Condition related | Other autoimmune conditions | 279.4x |
| IDIOPATHIC | All others with osteonecrosis but without any of the diagnoses listed above |  |
