# Supplementary material for: An underlying diagnosis of osteonecrosis of bone is associated with worse outcomes than osteoarthritis after total hip arthroplasty
Source: BMC Musculoskelet Disord. 2017 Jan 9;18:8. doi: 10.1186/s12891-016-1385-0 (PMC5223478; doi:10.1186/s12891-016-1385-0)
Supplement: Additional file 2: — Prevalence of Comorbidities by Primary Diagnosis at Total Hip Arthroplasty Surgery. This file shows the prevalence of various comorbidities in patients with osteoarthritis vs. osteonecrosis as the underlying reason and the primary diagnosis for the Total Hip Arthroplasty Surgery. (DOCX 20 kb) [file 12891_2016_1385_MOESM2_ESM.docx]

**Additional file 2.** Prevalence of Comorbidities by Primary Diagnosis at Total Hip Arthroplasty Surgery

|  | Total^1^ | | Osteoarthritis | | Osteonecrosis | | | Chi-Square P-Value |
| --- | --- | --- | --- | --- | --- | --- | --- | --- |
| Comorbidities | **n** | **%** | **n** | **%** | **n** | **%** | |  |
| Total | 17179 | 100.0 | 16509 | 100.0 | 670 | 100.0 |  | |
| Cases with at least one comorbidity | 14384 | 84.2 | 13818 | 84.2 | 566 | 84.7 |  | |
| Congestive heart failure | 480 | 2.8 | 455 | 2.8 | 25 | 3.7 | 0.206 | |
| Vascular disease | 671 | 3.9 | 651 | 4.0 | 20 | 3.0 | 0.279 | |
| Pulmonary circulation disease | 167 | 1.0 | 158 | 1.0 | 9 | 1.3 | 0.383 | |
| Peripheral vascular disease | 1005 | 5.9 | 969 | 5.9 | 36 | 5.4 | 0.536 | |
| Paralysis | 96 | 0.6 | 92 | 0.6 | 4 | 0.6 | 0.620 | |
| Other neurological disorders | 498 | 2.9 | 469 | 2.9 | 29 | 4.3 | 0.051 | |
| Chronic pulmonary disease | 2669 | 15.6 | 2504 | 15.3 | 165 | 24.7 | <0.001 | |
| Hypothyroidism | 2300 | 13.5 | 2226 | 13.6 | 74 | 11.1 | 0.114 | |
| Renal failure | 1567 | 9.2 | 1491 | 9.1 | 76 | 11.4 | 0.082 | |
| Liver disease | 443 | 2.6 | 391 | 2.4 | 52 | 7.8 | <0.001 | |
| Peptic ulcer disease x bleeding | 2 | 0.0 | 2 | 0.0 | 0 | 0.0 | 0.600 | |
| Acquired immune deficiency syndrome | 51 | 0.3 | 26 | 0.2 | 25 | 3.7 | <0.001 | |
| Lymphoma | 69 | 0.4 | 61 | 0.4 | 8 | 1.2 | 0.003 | |
| Metastatic cancer | 59 | 0.3 | 54 | 0.3 | 5 | 0.7 | 0.121 | |
| Solid tumor w/out metastasis | 205 | 1.2 | 198 | 1.2 | 7 | 1.0 | 0.584 | |
| Rheumatoid arthritis/collagen vascular disease | 505 | 3.0 | 468 | 2.9 | 37 | 5.5 | <0.001 | |
| Coagulopathy | 358 | 2.1 | 336 | 2.0 | 22 | 3.3 | 0.055 | |
| Weight loss | 146 | 0.9 | 135 | 0.8 | 11 | 1.6 | 0.047 | |
| Fluid and electrolyte disorders | 1065 | 6.2 | 1026 | 6.3 | 39 | 5.8 | 0.569 | |
| Chronic blood loss anemia | 441 | 2.6 | 430 | 2.6 | 11 | 1.6 | 0.186 | |
| Deficiency anemia | 2007 | 11.8 | 1900 | 11.6 | 107 | 16.0 | 0.001 | |
| Alcohol abuse | 466 | 2.7 | 401 | 2.4 | 65 | 9.7 | <0.001 | |
| Drug abuse | 229 | 1.3 | 203 | 1.2 | 26 | 3.9 | <0.001 | |
| Psychoses | 1223 | 7.2 | 1164 | 7.1 | 59 | 8.8 | 0.144 | |
| Depression | 992 | 5.8 | 945 | 5.8 | 47 | 7.0 | 0.239 | |
| Hypertension | 10336 | 60.5 | 9976 | 60.8 | 360 | 53.9 | 0.001 | |
| 1. Co-morbidities available for limited sample (operative years 2009-2012 and 3 geographical regions). Missing co-morbidities: N=99 (0.6%). Obesity (because of body mass index data) and diabetes (because of diabetes registry data) available in Table 1 for entire cohort and therefore not listed in this table. | | | | | | | | |
