# Supplementary material for: An underlying diagnosis of osteonecrosis of bone is associated with worse outcomes than osteoarthritis after total hip arthroplasty
Source: BMC Musculoskelet Disord. 2017 Jan 9;18:8. doi: 10.1186/s12891-016-1385-0 (PMC5223478; doi:10.1186/s12891-016-1385-0)
Supplement: Additional file 3: — Characteristics of patients with osteonecrosis by the underlying etiology, Idiopathic vs. Non-Idiopathic. This file shows the comparison of key characteristics between Idiopathic and Non-Idiopathic etiology of osteonecrosis. (DOCX 18 kb) [file 12891_2016_1385_MOESM3_ESM.docx]

**Additional file 3.** Characteristics of patients with osteonecrosis by the underlying etiology, Idiopathic vs. Non-Idiopathic

|  | | **Overall** | **Idiopathic** | **Non-Idiopathic** |
| --- | --- | --- | --- | --- |
| **~TOTAL~** | **N (%)** | **670 (100.0)** | **507 (75.7)** | **163 (24.3)** |
| Age, years | Median (IQR) | 56.0 (46.0-66.0) | 57.0 (48.0-66.0) | 55.0 (44.0-64.0) |
| Age category, years | <65 | 471 (70.3) | 347 (68.4) | 124 (76.1) |
|  | ≥65 | 199 (29.7) | 160 (31.6) | 39 (23.9) |
| Gender | Male | 383 (57.2) | 302 (59.6) | 81 (49.7) |
|  | Female | 287 (42.8) | 205 (40.4) | 82 (50.3) |
| Race | Asian | 62 (9.3) | 49 (9.7) | 13 (8.0) |
|  | Black | 106 (15.8) | 72 (14.2) | 34 (20.9) |
|  | White | 394 (58.8) | 308 (60.7) | 86 (52.8) |
|  | Hispanic | 93 (13.9) | 71 (14.0) | 22 (13.5) |
|  | Other/Multi | 14 (2.1) | 6 (1.2) | 8 (4.9) |
|  | Unknown | 1 (0.1) | 1 (0.2) | 0 (0.0) |
| BMI category, kg/m^2^ | <30 | 461 (68.8) | 337 (66.5) | 124 (76.1) |
|  | ≥30 and <35 | 127 (19.0) | 110 (21.7) | 17 (10.4) |
|  | ≥35 | 82 (12.2) | 60 (11.8) | 22 (13.5) |
| ASA category | 1 and 2 | 366 (54.6) | 297 (58.6) | 69 (42.3) |
|  | ≥3 | 303 (45.2) | 209 (41.2) | 94 (57.7) |
|  | [Missing] | 1 (0.1) | 1 (0.2) | 0 (0.0) |
| Diabetes | No | 550 (82.1) | 424 (83.6) | 126 (77.3) |
|  | Yes | 120 (17.9) | 83 (16.4) | 37 (22.7) |
