# Supplementary material for: An underlying diagnosis of osteonecrosis of bone is associated with worse outcomes than osteoarthritis after total hip arthroplasty
Source: BMC Musculoskelet Disord. 2017 Jan 9;18:8. doi: 10.1186/s12891-016-1385-0 (PMC5223478; doi:10.1186/s12891-016-1385-0)
Supplement: Additional file 4: — Patient characteristics for patients with Non-Idiopathic osteonecrosis only (Glucocorticoid-induced vs. Not). This file shows the comparison of key characteristics between Glucocorticoid-induced vs. Not glucocorticoid-induced among those with a non-Idiopathic etiology of osteonecrosis. (DOCX 16 kb) [file 12891_2016_1385_MOESM4_ESM.docx]

**Additional file 4.** Patient characteristics for patients with Non-Idiopathic osteonecrosis only (Glucocorticoid-induced vs. Not)

|  | | **Overall** | **Not Glucocorticoid-induced** | **Glucocorticoid-induced** |
| --- | --- | --- | --- | --- |
| ~TOTAL~ | N (%) | 163 (100.0) | 123 (75.5) | 40 (24.5) |
| Age, years | Median (IQR) | 55.0 (44.0-64.0) | 54.0 (44.0-62.0) | 61.0 (45.5-68.0) |
| Age category, years | <65 | 124 (76.1) | 97 (78.9) | 27 (67.5) |
|  | ≥65 | 39 (23.9) | 26 (21.1) | 13 (32.5) |
| Gender | Male | 81 (49.7) | 68 (55.3) | 13 (32.5) |
|  | Female | 82 (50.3) | 55 (44.7) | 27 (67.5) |
| Race | Asian | 13 (8.0) | 8 (6.5) | 5 (12.5) |
|  | Black | 34 (20.9) | 26 (21.1) | 8 (20.0) |
|  | White | 86 (52.8) | 64 (52.0) | 22 (55.0) |
|  | Hispanic | 22 (13.5) | 17 (13.8) | 5 (12.5) |
|  | Other/Multi | 8 (4.9) | 8 (6.5) | 0 (0.0) |
| BMI category, kg/m^2^ | <30 | 124 (76.1) | 96 (78.0) | 28 (70.0) |
|  | ≥30 and <35 | 17 (10.4) | 12 (9.8) | 5 (12.5) |
|  | ≥35 | 22 (13.5) | 15 (12.2) | 7 (17.5) |
| ASA category | 1 and 2 | 69 (42.3) | 60 (48.8) | 9 (22.5) |
|  | ≥3 | 94 (57.7) | 63 (51.2) | 31 (77.5) |
| Diabetes | No | 126 (77.3) | 99 (80.5) | 27 (67.5) |
|  | Yes | 37 (22.7) | 24 (19.5) | 13 (32.5) |
