# Supplementary material for: An underlying diagnosis of osteonecrosis of bone is associated with worse outcomes than osteoarthritis after total hip arthroplasty
Source: BMC Musculoskelet Disord. 2017 Jan 9;18:8. doi: 10.1186/s12891-016-1385-0 (PMC5223478; doi:10.1186/s12891-016-1385-0)
Supplement: Additional file 5: — Unadjusted outcomes in patients with osteonecrosis by the underlying etiology, Idiopathic vs. Non-Idiopathic. This file shows the unadjusted comparisons of the outcomes in patients with Idiopathic vs. Non-Idiopathic etiology of osteonecrosis. (DOCX 15 kb) [file 12891_2016_1385_MOESM5_ESM.docx]

**Additional file 5.** Unadjusted Outcomes in patients with osteonecrosis by the underlying etiology, Idiopathic vs. Non-Idiopathic

|  | | **Overall** | **Idiopathic** | **Non-Idiopathic** |
| --- | --- | --- | --- | --- |
| **~TOTAL~** | **N (%)** | **670 (100.0)** | **507 (75.7)** | **163 (24.3)** |
| Death, 90 days | | 4 (0.6) | 2 (0.4) | 2 (1.2) |
| Surgical site infection, any | | 7 (1.0) | 6 (1.2) | 1 (0.6) |
| Deep, 1-year | | 4 (0.6) | 3 (0.6) | 1 (0.6) |
| Superficial, 30-days | | 3 (0.4) | 3 (0.6) | 0 (0.0) |
| Venous thromboembolism, 90-days | | 10 (1.5) | 8 (1.6) | 2 (1.2) |
| Deep vein thrombosis | | 6 (0.9) | 4 (0.8) | 2 (1.2) |
| Pulmonary embolism | | 5 (0.7) | 5 (1.0) | 0 (0.0) |
| Readmission, 90 days unplanned | | 63 (9.4) | 39 (7.7) | 24 (14.7) |
| Revision, ever | | 11 (1.6) | 6 (1.2) | 5 (3.1) |
