# Supplementary material for: An underlying diagnosis of osteonecrosis of bone is associated with worse outcomes than osteoarthritis after total hip arthroplasty
Source: BMC Musculoskelet Disord. 2017 Jan 9;18:8. doi: 10.1186/s12891-016-1385-0 (PMC5223478; doi:10.1186/s12891-016-1385-0)
Supplement: Additional file 6: — Unadjusted outcomes in patients with Non-Idiopathic osteonecrosis only (Glucocorticoid-induced vs. Not Glucocorticoid-induced). This file shows the unadjusted comparisons of the outcomes in patients with Glucocorticoid-induced vs. Not Glucocorticoid-induced etiology of osteonecrosis among those with a non-Idiopathic etiology of osteonecrosis. (DOCX 14 kb) [file 12891_2016_1385_MOESM6_ESM.docx]

**Additional file 6.** Unadjusted outcomes in patients with Non-Idiopathic osteonecrosis only (Glucocorticoid-induced vs. Not Glucocorticoid-induced)

|  | | **Overall** | **Not Glucocorticoid-induced** | **Glucocorticoid-induced** |
| --- | --- | --- | --- | --- |
| ~TOTAL~ | N (%) | 163 (100.0) | 123 (75.5) | 40 (24.5) |
| Death, 90 days | | 2 (1.2) | 0 (0.0) | 2 (5.0) |
| Surgical site infection, any | | 1 (0.6) | 1 (0.8) | 0 (0.0) |
| Deep, 1-year | | 1 (0.6) | 1 (0.8) | 0 (0.0) |
| Venous thromboembolism, 90-days | | 2 (1.2) | 1 (0.8) | 1 (2.5) |
| Deep vein thrombosis | | 2 (1.2) | 1 (0.8) | 1 (2.5) |
| Readmission, 90 days unplanned | | 24 (14.7) | 17 (13.8) | 7 (17.5) |
| Revision, ever | | 5 (3.1) | 5 (4.1) | 0 (0.0) |
