# Supplementary material for: An underlying diagnosis of osteonecrosis of bone is associated with worse outcomes than osteoarthritis after total hip arthroplasty
Source: BMC Musculoskelet Disord. 2017 Jan 9;18:8. doi: 10.1186/s12891-016-1385-0 (PMC5223478; doi:10.1186/s12891-016-1385-0)
Supplement: Additional file 7: — Adjusted association of Non-Idiopathic vs. idiopathic cause of osteonecrosis with outcomes, revision and 90-day unplanned readmissions. This file shows the age-adjusted association of non-Idiopathic vs. idiopathic cause of osteonecrosis for revision and 90-day unplanned readmissions. (DOCX 14 kb) [file 12891_2016_1385_MOESM7_ESM.docx]

**Additional file 7.** Adjusted* association of Non-Idiopathic vs. idiopathic cause of osteonecrosis with outcomes, revision and 90-day unplanned readmissions

| **Non-Idiopathic vs. Idiopathic** | **Unadjusted OR (95%CI)** | **p-value** | **Adjusted OR (95%CI)** | **p-value** |
| --- | --- | --- | --- | --- |
| Readmission, 90 days unplanned | 2.07 (1.20 - 3.56) | 0.009 | 2.13 (1.23 - 3.68) | 0.007 |
|  | **Unadjusted HR (95%CI)** | **p-value** | **Adjusted HR (95%CI)** | **p-value** |
| Revision | 2.63 (0.80 - 8.69) | 0.112 | 2.52 (0.75 - 8.45) | 0.134 |

*Models were adjusted for age
