# Supplementary material for: An underlying diagnosis of osteonecrosis of bone is associated with worse outcomes than osteoarthritis after total hip arthroplasty
Source: BMC Musculoskelet Disord. 2017 Jan 9;18:8. doi: 10.1186/s12891-016-1385-0 (PMC5223478; doi:10.1186/s12891-016-1385-0)
Supplement: Additional file 8: — Adjusted* association of glucocorticoid-induced osteonecrosis vs. not glucocorticoid-induced osteonecrosis with outcomes. This file shows the age-adjusted association of glucocorticoid-induced osteonecrosis vs. not glucocorticoid-induced osteonecrosis for 90-day unplanned readmissions. (DOCX 14 kb) [file 12891_2016_1385_MOESM8_ESM.docx]

**Additional file 8**. Adjusted* association of glucocorticoid-induced osteonecrosis vs. not glucocorticoid-induced osteonecrosis with outcomes

| **Glucocorticoid-induced vs. Not** | **Unadjusted OR (95%CI)** | **p-value** | **Adjusted OR (95%CI)** | **p-value** |
| --- | --- | --- | --- | --- |
| Readmission, 90 days unplanned | 1.32 (0.50 - 3.46) | 0.569 | 1.17 (0.44 - 3.15) | 0.75 |
| *Model adjusted for age |  |  |  |  |
