# Supplementary material for: An underlying diagnosis of osteonecrosis of bone is associated with worse outcomes than osteoarthritis after total hip arthroplasty
Source: BMC Musculoskelet Disord. 2017 Jan 9;18:8. doi: 10.1186/s12891-016-1385-0 (PMC5223478; doi:10.1186/s12891-016-1385-0)
Supplement: Additional file 9: — Top 15 International Classification of Diseases, Ninth Revision (ICD-9) codes and descriptions of unplanned readmissions within 90 days of Total Hip Arthroplasty Surgery by primary diagnosis (osteoarthritis vs. osteonecrosis). This file shows the top 15 reasons for unplanned readmissions within 90 days of Total Hip Arthroplasty Surgery by primary diagnosis, osteoarthritis vs. osteonecrosis. (DOCX 17 kb) [file 12891_2016_1385_MOESM9_ESM.docx]

**Additional file 9.** Top 15 International Classification of Diseases, Ninth Revision (ICD-9) codes and descriptions of unplanned readmissions within 90 days of Total Hip Arthroplasty Surgery by primary diagnosis (osteoarthritis vs. osteonecrosis)

|  | | | | |
| --- | --- | --- | --- | --- |
| ICD9 Code | **ICD9 Diagnosis Description** | **n** | | **%** |
| Osteoarthritis cohort: n=16,509 (96.0%), 1414 readmissions occurred in 846 patients | | **1414** | | **100%** |
| 996.42 | Dislocation of prosthetic joint | 100 | | 7.1% |
| 996.66 | Infection and inflammation reaction due to joint prosthesis | 74 | | 5.2% |
| 998.59 | Other postoperative infection | 53 | | 3.7% |
| 38.9 | Unspecified septicemia | 50 | | 3.5% |
| 996.44 | Periprosthetic fracture around prosthetic joint | 31 | | 2.2% |
| 719.45 | Pain in joint pelvic region and thigh | 30 | | 2.1% |
| 415.19 | Other pulmonary embolism and infarction | 29 | | 2.1% |
| 8.45 | Intestinal infections due clostridium difficile | 28 | | 2.0% |
| 998.12 | Hematoma complicating a procedure | 26 | | 1.8% |
| 599 | Urinary tract infection site not specified | 26 | | 1.8% |
| 410.71 | Acute MI subendocardial infarction, initial episode of care | 21 | | 1.5% |
| 682.6 | Cellulitis and abscess of leg except foot | 21 | | 1.5% |
| 996.77 | Other complications due internal joint prosthesis | 18 | | 1.3% |
| 486 | Pneumonia, organism unspecified | 18 | | 1.3% |
| 584.9 | Acute kidney failure unspecified | 16 | | 1.1% |
| Osteonecrosis cohort: n=670 (4.0%), 119 readmissions occurred in 64 patients | | **119** | | **100%** |
| 996.42 | Dislocation of prosthetic joint | 13 | | 10.9% |
| 38.9 | Unspecified septicemia | 9 | | 7.6% |
| 486 | Pneumonia, organism unspecified | 6 | | 5.0% |
| 282.62 | Sickle cell anemia with crisis | 5 | | 4.2% |
| 493.22 | Chronic obstructive asthma with exacerbation | 4 | | 3.4% |
| 998.59 | Other postoperative infection | 3 | | 2.5% |
| 38.42 | Septicemia due to Escherichia coli | 3 | | 2.5% |
| 786.5 | Chest pain unspecified | 2 | | 1.7% |
| 821.01 | Closed fracture of shaft of femur | 2 | | 1.7% |
| 414.01 | Coronary atherosclerosis native coronary artery | 2 | | 1.7% |
| 345.2 | Epileptic petit mal status | 2 | | 1.7% |
| 996.66 | Infection and inflammatory reaction due to internal joint prosthesis | | 2 | 1.7% |
| 8.45 | Intestinal infections due clostridium difficile | 2 | | 1.7% |
| V57.89 | Other specified rehabilitation procedure other | 2 | | 1.7% |
| 298.9 | Unspecified psychosis | 2 | | 1.7% |
